# The Non-Panel Movement

**Published:** 1913-10-25

**Authors:** 


### THE NON-PANEL MOVEMENT.

As recently as last week, in discussing the possibilities which the next two or three years may hold in store for panel practitioners in respect of remuneration, we quoted the example of Germany and the legitimate dissatisfaction which exists among the doctors there concerning their treatment under the German Insurance Acts, the acknowledged prototype of our own. By a coincidence last week's issue of the *British Medical Journal* contains an account, from a special correspondent in Berlin, of certain alterations and amendments in the law to come in force on January 1, 1914, which confirm very amply the tone of our own warnings. The Legislature, says this article, has once more turned a deaf ear to the claims and suggestions put forward by the medical profession; and it proceeds to make it clear that the net result of the changes will be to give more power than before to the organisations which correspond to the approved societies of the British Act. It is probable that the reorganisation in question will considerably curtail the present powers of the administrative bodies, and that the sad but common spectacle—in Berlin especially—of medical practitioners obtaining their insurance appointments by personal canvass and other undignified means will now become a rarity. In this respect, therefore, improvement may be looked for; and certainly the power which these bodies have, and exercise, over the medical profession has been a long-standing blot on German insurance.

In other ways the new regulations are little calculated to improve the lot of the panel practitioner. The income limit, for instance, of those compelled to insure has been raised from £100 to £125 a year, and all those are also included who work under contract, whether at home or in workshops, up to an income of £250. Furthermore, artificers, technical workers, school teachers, tutors, foremen, shop assistants, and those connected in any capacity with the stage are now included, provided their remuneration is less than £125. In the event of their circumstances improving they may still remain voluntary contributors until their income exceeds £200. It will be seen that in these details the changes in Germany tend to approximate the law there towards the provisions of our own Act; in other words, to include among the insured larger and larger numbers of those who have hitherto been paying private patients. It is computed that an additional nine millions of the population will be brought within the provisions of insurance, and that in some districts 92 to 95 per cent. of the population will be insured. A bright prospect for private practice in Germany certainly!

In spite of the most urgent representations by the medical profession there is no indication of any increase in the capitation fee, which is five shillings per patient per annum, and compares unfavourably with the (present) British figure. It will be remembered, however, that this sum is a reduction on the original sum, and we trust that the lesson and the moral we pointed last week *apropos* of this fact have not been forgotten.

If any further evidence is required as to the working of the German Acts, the very telling exposure of their defects and their working which we print in another column (page 97) should surely suffice. But for the moment the point we would here urge for consideration is the future prospects of panel practice in the light, first, of our own Act and the tendencies of those who control and direct its operations, and, secondly, of the manner in which its exemplar in Germany has already affected the position of the medical profession in that country. At the moment the position of the British profession is probably less unfavourable than that of their German brethren; but the disunion and consequent overthrow of last January do not promise well for defence against future aggression. In addition, there is evidence that a reaction against panel practice is setting in, more so in the towns than in the country. The letter of a correspondent from the North of England, which appears on page 99, may usefully be read by any who doubt the truth of this statement, for the medical man in question is one in whose veracity and powers of observation we place full confidence, though we are not at liberty to give his name.

Another interesting contribution of the week to the literature of the non-panel movement is Dr. Buttar's *apologia pro vita sua* in last week's *British Medical Journal*. Dr. Buttar is, and has long been, one of those who object to the present form of panel practice. Because he does not go far enough to please the most extreme section of the anti-panel movement he seems to have incurred almost as much dislike among them as the author of the Act itself. On the other hand, those members of the British Medical Association who regard the Act and all its regulations with favour are equally hostile to Dr. Buttar, whose criticisms they have had to endure. In the letter which this gentleman has published he outlines a comprehensive policy which seems to us in most details sane and rational. His proposals are supported by arguments which will appeal to extremists on neither side, but which all moderate men will, we think, recognise as full of sound sense. They are as follows:—

(1) The substitution of the medical register for the panel.

(2) The removal of all control of medical practice from lay bodies and the restoration of all disciplinary power to the General Medical Council with altered constitution and wider powers.

(3) The full recognition of local medical committees to deal with all arrangements for the medical treatment of insured persons in each area, while the Insurance Committees watch over the financial conditions and the interests of the insured persons under their charge.

(4) Proper development of Section 15 (3) of the Act, allowing every suitable person to make his own arrangements, without any such documents as 43/I.C.

(5) Recognition by the medical profession that while contract practice is bad, in certain areas and certain cases contract practice is unavoidable. That for this reason contract practice should be kept within the narrowest possible limits until such time as it may be possible to abolish it altogether.

(6) Realisation that friendly societies are really admirable institutions, and that friendly society *control* is the true enemy. That one strong Local Medical Committee is a great deal more powerful than a dozen separate friendly societies in the same area, while 100 or 200 Local Medical Committees are as nothing against a single bureaucratic Insurance Commission. The realisation of these facts may help the profession greatly to be prepared in case the friendly societies ever succeed in regaining control of medical benefit.

We may doubt whether Dr. Buttar's scheme is practicable in the immediate future, but no one can deny that it would vastly improve both the profession of medicine and the administration of medical benefits for the advantage of the insured.

